# Mitochondrial DNA Variation of Leber’s Hereditary Optic Neuropathy in Western Siberia

**DOI:** 10.3390/cells8121574

**Published:** 2019-12-04

**Authors:** Elena Starikovskaya, Sofia Shalaurova, Stanislav Dryomov, Azhar Nazhmidenova, Natalia Volodko, Igor Bychkov, Ilia Mazunin, Rem Sukernik

**Affiliations:** 1 Laboratory of Human Molecular Genetics, Institute of Molecular and Cellular Biology, SBRAS, Novosibirsk 630090, Russiastasundr@gmail.com (S.D.); deviliona@yandex.ru (A.N.); sukernik@gmail.com (R.S.); 2 Department of Pediatrics, University of Alberta, Edmonton, AB T6G 2R3, Canada; 3 Novosibirsk Branch of S.N. Fedorov NMRC “MNTK Eye Microsurgery”, Moscow 127486, Russia; 4 Center of Life Sciences, Skolkovo Institute of Science and Technology, Skolkovo 121205, Russia

**Keywords:** LHON, Siberian population, ancient mutation, specific genetic background

## Abstract

Our data first represent the variety of Leber’s hereditary optic neuropathy (LHON) mutations in Western Siberia. LHON is a disorder caused by pathogenic mutations in the mitochondrial DNA (mtDNA), inherited maternally and presents mainly in young adults, predominantly males. Clinically, LHON manifests itself as painless central vision loss, resulting in early onset of disability. The epidemiology of LHON has not been fully investigated yet. In this study, we report 44 genetically unrelated families with LHON manifestation. We performed whole mtDNA genome sequencing and provided genealogical and molecular genetic data on mutations and haplogroup background of LHON patients. Known “primary” pathogenic mtDNA mutations (MITOMAP) were found in 32 families: m.11778G>A represents 53.10% (17/32), m.3460G>A—21.90% (7/32), m.14484T>C–18.75% (6/32), and rare m.10663T>C and m.3635G>A represent 6.25% (2/32). We describe potentially pathogenic m.4659G>A in one subject without known pathogenic mutations, and potentially pathogenic m.6261G>A, m.8412T>C, m.8551T>C, m.9444C>T, m.9921G>A, and m.15077G>A in families with known pathogenic mutations confirmed. We suppose these mutations could contribute to the pathogenesis of optic neuropathy development. Our results indicate that haplogroup affiliation and mutational spectrum of the Western Siberian LHON cohort substantially deviate from those of European populations.

## 1. Introduction

Leber’s hereditary optic neuropathy (LHON) is a form of hereditary disorder caused by pathogenic mutations in mitochondrial DNA. These mutations are non-synonymous, and affect genes coding for different subunits of complex I of the mitochondrial respiratory chain. The occurrence of such kinds of mutations in mtDNA subunits leads to dysfunction of the electron transport, increased reactive oxygen species production, and defective ATP synthesis [1,2,3]. Retinal ganglion cells are highly susceptible to death during LHON progression, because of their high sensibility to disrupted ATP production and oxidative stress [4]. Therefore, LHON is usually painless, acute, or subacute, central visual loss of one or both eyes, which results in early onset of disability. The onset of Leber’s hereditary optic neuropathy is relatively rare and has better visual prognosis. The peak age of onset of visual loss among LHON carriers is 20–30 years old [5]. In some cases, LHON patients have been reported as having additional neurologic, cardiac, and endocrine disorders [6,7,8]. Leber’s disease is maternally inherited and manifests itself in youth predominantly (~50% of man and ~10% of women). Interestingly, this sex predilection cannot be explained by the principles of mitochondrial inheritance [4]. 

At the present time, a number of mtDNA point mutations have been described, but the most prevalent are m.3460G>A, m.11778G>A, and m.14484T>C, accounting for about 90% of cases of LHON worldwide. The prevalence of each mutation varies among different populations, but the average is 69–92% for m.11778G>A, and 3–19% for m.14484T>C and m.3460G>A [9,10,11,12,13,14]. However, there are significant deviations from the average in some populations, for example, among French Canadians 87% of cases are due to m.14484T>C as a result of a founder effect [15]. Moreover, phenotypic expression of these primary mutations has been found to vary in different populations and different pedigrees. This incomplete penetrance suggests that other factors, such as mtDNA haplogroup background, nuclear genetic background, and environmental factors, may influence the modulation of phenotypic expression and severity of the disease [16,17,18].

Consequently, the worldwide prevalence of LHON varies in different populations and is unknown for the majority of them. This prevalence is estimated to range between 1:30,000 and 1:50,000 [19,20]. The epidemiology of LHON has not been fully investigated in Russian Federation, and our previous studies included the description of isolated cases [21,22,23]. Hence, in the present study, we report 44 genetically unrelated LHON families, performed whole mtDNA sequencing, and provide molecular genetic data on mutations and the haplogroup background of LHON patients in the Western Siberian population.

## 2. Materials and Methods

### 2.1. Subjects

This study was approved by the Ethics Committee IRB 00001360 affiliated with Vector State Research Center of Virology and Biotechnology (SRC VB Vector), Novosibirsk, Russian Federation. The total number of subjects in the study is 168 individuals from 44 unrelated families (85 affected and 83 healthy carriers), including 17 cases from our previous studies [21,22,23]. The clinical follow-up of LHON patients has been carried out by the Novosibirsk Branch of Federal Eye Microsurgery Department since 1997, conducted by one of the authors. The clinical diagnosis was based on a combination of symptoms and signs: painless acute or subacute central vision loss; fundus changes; and visual field abnormality, such as pseudopapilledema, optic nerve atrophy, and central or centrocecal scotoma. All the individuals made an informed decision to take part in the study and provided written consent. Family history was taken in each case to identify maternal inheritance of symptoms. The complete mtDNA genomes were sequenced for the family’s probands, and for the other individuals the certain mutations were confirmed by sequencing of associated mtDNA regions.

### 2.2. MtDNA Analysis

Whole peripheral blood samples were collected from the donors in 10 mL Vacutech EDTA tubes. Total DNA was extracted from a buffy-coat layer using the SileksMagNA-G Blood DNA Isolation kit, according to the manufacturer’s protocols. The complete sequencing procedure entailed PCR amplification of 22 overlapping mtDNA templates [24], which were sequenced in both directions with BigDye 3.1 terminator chemistry (PE Applied Biosystems, Foster City, CA, United States). The trace files were analyzed with Sequencher (version 4.5 GeneCode Corporation) software. To perform capillary electrophoresis on an ABI Prism 3130XL DNA Analyzer, we used core facilities of the “Genomika” Sequencing Center (SBRAS, Novosibirsk, Russian Federation). Variants were scored relative to the Reconstructed Sapiens Reference Sequence, RSRS [25]. MtDNA haplotypes were identified following the nomenclature suggested by the PhyloTree Build 17 [26]. Forty-two mitochondrial genomes obtained through this study were deposited in GenBank with accession numbers MN413201–MN413242. Two genomes, EU807741.1 and EU807742.1, had been deposited to the GenBank earlier [21].

### 2.3. Penetrance Analysis

We determined penetrance as the proportion of affected individuals from all maternally related family members using family pedigrees [19]. Values for both men and women were calculated separately.

### 2.4. Analysis of Pathogenicity for Non-Synonymous Mutations

To make sure that the revealed non-synonymous mtDNA mutations were not sequencing errors or to not point out to general population polymorphisms, and in turn to find out the disease-associated polymorphisms among those published earlier, we used several databases: MITOMAP [27]; mtDB (Human Mitochondrial Genome Database, containing 2704 human mitochondrial genomes) [28]; and HmtDB (Human Mitochondrial DataBase), which contains 32922 human mitochondrial genomes [29]. To assess the possible pathogenicity of these mutations, and to predict whether a protein sequence variation affects protein function, we used the following web applications: MutPred 1.2 [30], MutPred 2 [31], PolyPhen–2 [32], PROVEAN (Protein Variation Effect Analyzer) [33], and SIFT (Sorting Intolerant from Tolerant) [34]. All the sources are provided in the public domain. 

## 3. Results

From 44 LHON families, 32 harbored a primary mutation; the results are shown in Table 1. Among families with a primary mutation, the m.11778G>A represented 53.10% (17/32), m.3460G>A was 21.90% (7/32), and m.14484T>C represented18,75% (6/32). Rare m.10663T>C and m.3635G>A represented 6.25% (2/32) of the families.

According to the family pedigrees, only 50% (22/44) of cases had a family history of vision loss in the maternal lineage in more than one generation, among which m.11778G>A represented 36% (8/22), m.3460G>A covered 23% (5/22), and the m.14484T>C was 14% (3/22), as well as those without primary mutations (LHON-like cases), which represented 18% (4/22). Rare mutation cases (m.10663T>C and m.3635G>A) were family–inherited. In total, 39% (17/44) of cases were sporadic, among which 13 were cases with only one affected person diagnosed, and four were cases with two affected persons in one generation. Among the sporadic cases, m.11778G>A represented 41% (7/17), m.3460G>A was 12% (2/17), m.14484T>C was 6% (1/17), and the LHON-like cases represented 41% (7/17) of the total.

Summary information on penetrance is shown in Table 2. The penetrance is highly variable between separate families, even with the same primary mutation. The average penetrance among men was 32% (6–100%) and 12% among women (0–58%); these correlate with data previously published [4]. However, there are some families with higher penetrance among females than among males: L24, L26, and L28. 

In 12 families with clear-cut LHON phenotypes, no pathogenic mtDNA mutations were found. Analysis of the mtDNA revealed non-synonymous mutations: m.4766A>G, m.13105A>G, m.14002A>G, which have not been noted as associated with LHON or other diseases in the MITOMAP database. All pathogenicity prediction tools indicated low probability that the amino acid substitutions are disease-associated for these mutations. Mutation m.4659G>A has been previously reported as being associated with Parkinson’s disease [35], as well as in an Australian LHON pedigree that was heteroplasmic for the m.14484T>C [36]. Polyphen-2 predicted the pathogenicity for this mutation as benign and MutPed 2 showed low probability score, but MutPred 1.2, PROVEAN, and SIFT determined this mutation as deleterious. The results are shown in Table 3. 

In several families with primary mutations (L01, L03, L12, L28, L30, L40, L43, and L50) we found out additional, non-synonymous mutations (Table 4). Mutations m.8875T>C, m.14582A>G, m.8400T>C, and m.4639T>C were neutral, and mutation m.9444C>T had a high probability of being pathogenic, according to data from all the pathogenicity prediction tools; for other mutations, we observed divergence of prediction results. Since prediction results for primary pathogenic mutations diverged too (see Table 3), novel non-synonymous nucleotide change was considered potentially pathogenic if it had extremely low frequency in the general population, and if it was predicted by at least three algorithms to have an effect on protein function. For mutations m.6261G>A and m.15468C>T, only PolyPhen2 predicted pathogenicity as probably damaging and possible damaging, respectively. However, mutation m.6261G>A had already been reported by Abu-Amero [37] in patients with optic neuropathy, and also as a somatic mutation associated with prostate cancer. Interestingly, the family (L01) with m.6261G>A and m.11778G>A has the same haplogroup, T2, as the case reported by Abu-Amero. Other mutations (m.8412T>C, m.8551T>C, m.9921G>A, m.15077G>A) were predicted as pathogenic by at least by three algorithms, but the first three of them have not been noted as associated with diseases in the MITOMAP database, and mutation m.15077G>A was reported as being associated with maternally-inherited isolated deafness [38].

**Figure 1:**
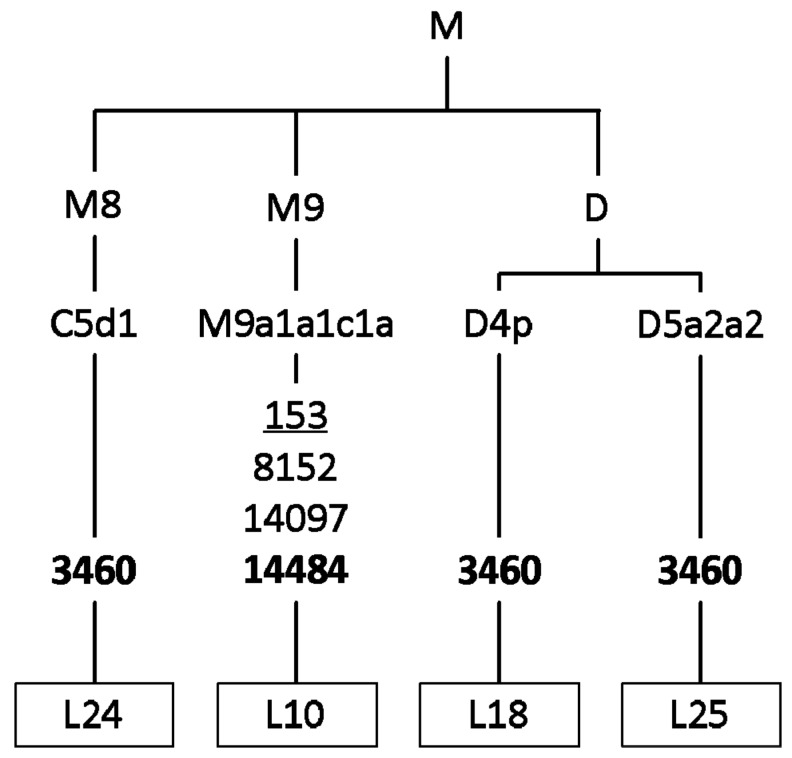
Phylogenetic tree based on the complete mtDNA genome sequences of pedigree probands with pathogenic LHON mutations (M8, M9, and D haploclusters). The non-synonymous coding region variants are denoted by “ns” (known pathogenic mutations designated in bold). Mutations are transitions unless a specific base change was specified; deletions are denoted by “del”; underlined mutations are recurrent.

**Figure 2:**
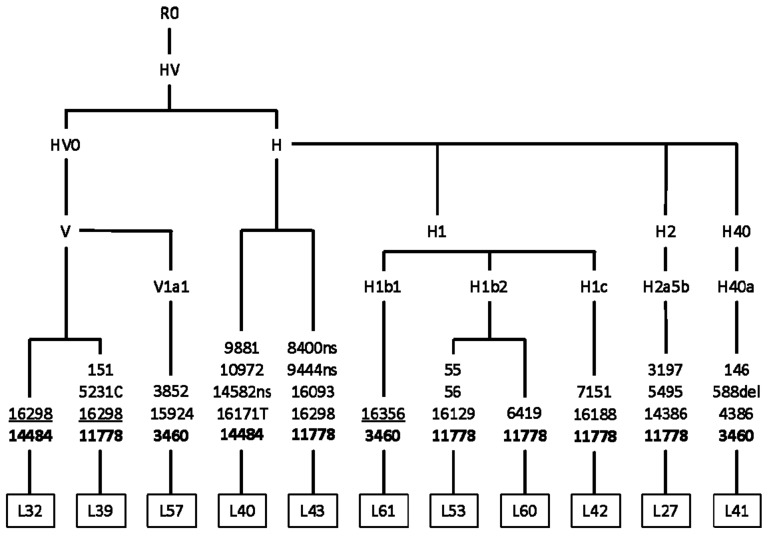
Phylogenetic tree based on the complete mtDNA genome sequences of pedigree probands with pathogenic LHON mutations (H’V haplocluster). The non-synonymous coding region variants are denoted by “ns” (known pathogenic mutations designated in bold). Mutations are transitions unless a specific base change was specified; deletions are denoted by “del”; underlined mutations are recurrent.

**Figure 3:**
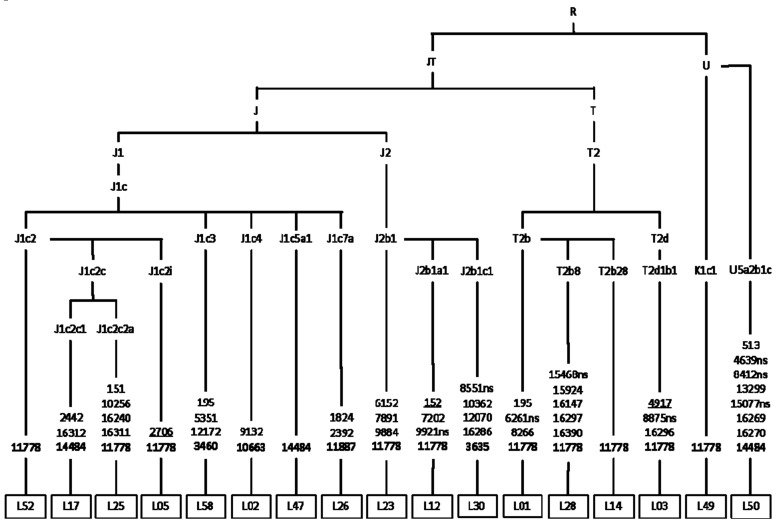
Phylogenetic tree based on the complete mtDNA genome sequences of pedigree probands with pathogenic LHON mutations (J’T, and U’K haploclusters). The non-synonymous coding region variants are denoted by “ns” (known pathogenic mutations designated in bold). Mutations are transitions unless a specific base change was specified; deletions are denoted by “del”; underlined mutations are recurrent.

Phylogenetic analysis illustrates that Siberian carriers of pathogenic LHON mutations are unrelated and belong to different maternal lines. In rare cases (4/44), m.3460G>A and m.14484T>C belong to East Eurasian M8, M9, and D haploclusters (Figure 1). The classic (m.3460G>A, m.11778G>A, m.14484T>C) and rare LHON-causing mutations occur mostly in the mtDNA background of West Eurasian haploclusters H’V (Figure 2), J’T and U’K (Figure 3).

## 4. Discussion

### 4.1. Leber’s Hereditary Optic Neuropathy Primary Mutations

Regarding our preliminary data, the frequencies of primary mutations are different from frequencies reported for Europe and Asia. The most prevalent m.11778G>A (~55%) is less common in Western Siberia than in Europe, at ~69% [9]; as well as in China and Japan, at ~90% [10,13]. The prevalence of m.3460G>A (~24%) is twice as much as in Europe, but m.14484T>C (~14%) do not deviate from those of other European populations [9].

In 12 families with LHON-like manifestation, no known pathogenic mtDNA mutation was found. However, there are other elaborations in which the clinical diagnosis cannot be confirmed by molecular genetic analysis [37,39,40,41]. Definitively, mtDNA mutation-caused LHON is clinically indistinguishable from the other forms of optic neuropathy, such as a dominant optic neuropathy (DOA), especially when it is sporadic. Compared to LHON, DOA visual loss is detected between ages 4 and 6 in the majority of patients, and 58–84% of patients with DOA report visual impairment by age 11 [42]. In our 12 cases, the ages of onset were between 13–36 years. We will be studying these cases for the mutation spectrum of common pathogenic genes for DOA in future.

### 4.2. Penetrance

We suppose that relatively reduced incidence of LHON in Western Siberia is associated with incomplete penetrance and diagnostic difficulties of atypical (e.g., late-onset) and combined (e.g., multiple sclerosis) forms of LHON. Patients with LHON could have also been wrongly diagnosed as suffering from toxic amblyopia, tobacco–alcohol amblyopia, or optic neuritis [41]. On the other hand, the problem is that patients do not know their family history. Molecular testing for LHON is not routinely performed in patients with optic atrophy in Russian Federation. Identification and registration of unaffected carriers plays an important role for prevention of disease manifestation. For example, there is strong evidence that smoking is associated with an increased risk of visual failure among LHON carriers—93% penetrance of vision loss in male smokers versus 66% in male non-smokers [17].

Our observations highlight the importance of molecular genetic examination for unaffected carriers. The presence of pathogenic mutations should be tested not only for probands, but for all relatives in the maternal line, Since the proportion of sporadic cases is about 40% according to our published data [4].

### 4.3. Potentially Pathogenic Mutation m.4659G>A

We found m.4659G>A in one subject without any known primary mutations (L51). This sequence change is located at codon 64 in the functional domain of the ND2 gene and changes an alanine—a hydrophobic amino acid—into threonine—a neutral amino acid. Mutation m.4659G>A has been reported as being associated with LHON in an Australian pedigree that also had heteroplasmic mutations m.14484T>C and m.5460G>A [36]. This family had 10 maternally-related descendants, five of whom had vision loss. Unfortunately, our patient does not know his family history, and we could not confirm maternal inheritance for this mutation. However, m.4659G>A has very low frequency in the general population (0.0011–0.00161), and has a high probability of being pathogenic (Table 3).

### 4.4. Additional Non-Synonymous Mutations Revealed in LHON Cases

The phenomenon of co-existence of two pathogenic mutations in one family has already been described. The first case included m.4659G>A, m.5460G>A, and m.14484T>C in an Australian LHON pedigree, described above [36]. In the second case, a Polish family harboring two primary LHON mutations m.3460G>A and m.11778G>A occurred in a haplogroup H background [43]. In the third case, a family harbored two primary LHON mutations, m.11778G>A and m.14484T>C, and both mutations had a synergistic pathogenic effect on protein function, as well as a higher degree of heteroplasmy of the m.14484T>C, correlated with an earlier age at onset [44]. Finally, the fourth example is a unique double-mutant ND4L with two concurrent mutations (m.10609T>C and m.10663T>C) in an Arab pedigree from Kuwait [45]. 

We reported mutations m.6261G>A, m.8412T>C, m.8551T>C, m.9444C>T, m.9921G>A, and m.15077G>A, which could be potentially pathogenic because of their low frequency in the general population, and high probability of pathogenicity according to data from different prediction tools. Two of them, m.6261G>A and m.15077G>A, have already been reported in subjects with optic neuropathy and maternally-inherited isolated deafness, respectively. However, we suppose that additional non-synonymous mutations could either have a synergistically pathogenic or a protective effect. To demonstrate the full significance of novel mutations, a respiratory chain assay would need to be performed. An example is the study [38], where cybrids with m.15077G>A showed normal activities for mitochondrial electron chain enzymatic complexes.

### 4.5. Haplogroup Analysis

Our LHON cohort from the Western Siberia region is represented predominantly by West Eurasian haplogroups and includes several East Eurasian haplogroups, namely C5d1 (L24), D4p (L18), D5a2a2 (L25), and M9a1a1c1a (L10).

Rare LHON mutations m.10663T>C and m.3635G>A were found in Russian families from Kazakhstan (the first) and the Novosibirsk region (the second), associated with the European haplogroups J1c4 and J2b1c1, respectively [21,22]. Mutation m.10663T>C was also reported in the background of the haplogroups J1c2c, L2a1, L3’4, and L3f1b [37,45,46,47], and mutation m.3635G>A was reported in haplogroups R11a, D4g2b, M7b4, F1a, B5b, and M7b [48,49]. The presence of the same pathogenic mutations on the background of various mitochondrial haplogroups confirms that pathogenic LHON mutations arise de novo, independently from the mtDNA or ethnic backgrounds.

It is known that the clinical impact of mDNA mutations may be modulated by mitochondrial haplogroup background. For example, Hudson et al. performed a multicenter study of 3613 subjects from 159 different families, and showed that the risk of visual failure is greater when m.11778G>A or m.14484T>C mutations are present in specific subgroups of haplogroup J; the same as the m.3460G>A mutation is present in haplogroup K, and the risk of visual failure is significantly lower when m.11778G>A occurs in haplogroup H [50]. Romero et al. supposed that haplogroup D has a protective effect in carriers of LHON mutations. His hypothesis was based on the fact that there was a markedly decreased frequency of haplogroup D in Chilean subjects with LHON, as haplogroup D is one of the most common in the Chilean population [12]. Also, other experimental research serves as proof that cybrids and fibroblasts bearing LHON mutations have different response to neurotoxic agents, depending on haplogroup background [51].

It has been suggested that at the end of the last glaciation, phylogenetically more ancient mutations could have provided their carriers with adaptive advantages during the human population expansion. Today, those mutations contribute to the saving and expression of weakly pathogenic LHON mutations, which appear randomly in different region-specific genetic backgrounds [52,53]. The theory could be tested by further searching of pathogenic, LHON-causing mutations in relation to specific mtDNA backgrounds (phylogenetically ancient set of mutations).

New data collected from future studies regarding mtDNA variations of LHON in Western Siberia might be used to develop a LHON system registry in the Russian Federation. We intend to conduct consecutive experimental research, including the parameters of the pathogenicity of each novel substitution.

## 5. Limitations of the Study

The main limitation of the data presented is the absence of physiological tests as proof with respect to the pathogenicity of novel mtDNA substitutions. Additional tests should be done, such as oxygen consumption, ATP and ROS measuring, and electron microscopy study (for example, [54]). In addition, development of the newest editing systems [55] could give us more reliable instruments to test pathogenicity.

## Figures and Tables

**Table 1:** Summary data for examined Leber’s hereditary optic neuropathy (LHON) and LHON-like (without primary mutations) families. Age of onset of visual loss vary between families/patients; the peak age of onset is ~20–30 years old. Some families were published previously * [23]; ** [22]; *** [21].

No.	Family Name	Ethnicity in Maternal Line	Number of Examined Individuals (Affected/Healthy)	Family History of Visual Loss	Primary LHON Mutation (MITOMAP)	mtDNA Haplogroup
1	L18 *	Altaian	4 (2/2)	No	m.3460G>A	D4p
2	L24 *	Tuvinian	19 (7/12)	Yes	m.3460G>A	C5d1
3	L25 * * *	Russian	5 (3/2)	Yes	m.3460G>A	D5a2a2
4	L41	German	2 (1/1)	No	m.3460G>A	H40a
5	L57	-/-	1 (1/0)	Yes	m.3460G>A	V1a1
6	L58	-/-	2 (1/1)	Yes	m.3460G>A	J1c3
7	L61	-/-	4 (2/2)	Yes	m.3460G>A	H1b1
8	L30 * * *	-/-	19 (11/8)	Yes	m.3635G>A	J2b1c1
9	L2 * *	-/-	6 (2/4)	Yes	m.10663T>C	J1c4
10	L1 *	Russian	9 (1/8)	Yes	m.11778G>A	T2b
11	L3 *	-/-	4 (1/3)	Yes	m.11778G>A	T2d1b1
12	L5 *	-/-	1 (1/0)	Yes	m.11778G>A	J1c2i
13	L12 *	-/-	2 (1/1)	No	m.11778G>A	J2b1a1
14	L14 *	-/-	5 (2/3)	No	m.11778G>A	T2b28
15	L23 *	Azerbaijani	1 (1/0)	Unknown	m.11778G>A	J2b1
16	L26 *	-/-	13 (8/5)	Yes	m.11778G>A	J1c7a
17	L27 * * *	-/-	11 (2/9)	Yes	m.11778G>A	H2a5b
18	L28 * * *	-/-	9 (2/7)	Yes	m.11778G>A	T2b8
19	L38	Ukrainian	2 (2/0)	Yes	m.11778G>A	J1c2c2a
20	L39	Unknown	2 (1/1)	No	m.11778G>A	V
21	L42	Belarusian	2 (1/1)	Yes	m.11778G>A	H1c
22	L43	Russian	3 (1/2)	No	m.11778G>A	H
23	L49	Unknown	1 (1/0)	No	m.11778G>A	K1c
24	L52	Russian	3 (1/2)	No	m.11778G>A	J1c2
25	L53	Unknown	3 (1/2)	Unknown	m.11778G>A	H1b2
26	L60	Russian	2 (1/1)	No	m.11778G>A	H1b2
27	L10 *	-/-	8 (3/5)	Yes	m.14484T>C	M9a1a1c1a
28	L17 *	-/-	8 (4/4)	Yes	m.14484T>C	J1c2c1
29	L32	Unknown	1 (1/0)	Unknown	m.14484T>C	V
30	L40	Albanian	1 (1/0)	Yes	m.14484T>C	H
31	L47	-/-	1 (1/0)	No	m.14484T>C	J1c5a1
32	L50	Unknown	1 (1/0)	Unknown	m.14484T>C	U5a2b1c
33	L6	-/-	2 (2/0)	No	-	U4a1d
34	L8	Unknown	1 (1/0)	No	-	U2e1
35	L9	Russian	2 (2/0)	Yes	-	U5a1b1c1
36	L20	-/-	3 (2/1)	Yes	-	U4b1b1
37	L31 * * *	-/-	3 (2\1)	Yes	-	U3b1b
38	L45	-/-	2 (1/1)	No	-	U5a2e
39	L46	-/-	2 (1/1)	No	-	H13a1d
40	L51	Unknown	1 (1/0)	Unknown	-	U2c1b
41	L54	Russian	2 (1/1)	No	-	U4a2a
42	L56	-/-	2 (1/1)	No	-	J1c1b1
43	L59	Ukrainian	2 (1/1)	No	-	V7a
44	L62	-/-	1 (1/0)	Yes	-	H

**Table 2:** Summary information about penetrance.

	m.11778G>A (*n* = 15)	m.14484T>C (*n* = 4)	m.3460G>A (*n*= 7)	Average
Males	34%	46%	15%	32%
Females	12%	12%	10%	12%

**Table 3:** Non-synonymous mutations revealed in LHON-like cases. Known primary mutations (m.3460G>A, m.3635G>, m.10663T>C, m.11778G>A, and m.14484T>C) are placed in bold to demonstrate distinction between different prediction algorithms and frequencies in general population for pathogenic mutations. A MutPred 1.2 score > 0.75 and a Mutpred 2 score > 0.50 would suggest pathogenicity.

Mutation	Protein-Coding Region of mtDNA	Amino Acid Substitution	PolyPhen – 2 Score	MutPred 1.2/2 Score (Cutoff 0.75/0.50)	PROVEAN/SIFT Pathogenicity Prediction	Frequency in General Population (as per mtDB)	Frequency in General Population (as per HmtDB)	Family
m.14002A>G	ND5	T556A	0.002 (benign)	0.387/0.059	Neutral/Tolerated	0.0037	0.00289	L45
m.4766A>G	ND2	M99I	0.001 (benign)	0.571/0.225	Neutral/Tolerated	0	0.00009	L46
m.4659G>A	ND2	A64T	0.029 (benign)	0.790/0.256	Deleterious/Damaging	0.0011	0.00161	L51
m.13105A>G	ND5	I257V	0.001 (benign)	0.198/0.032	Neutral/Tolerated	0.0612	0	
**m.3460G>A**	ND1	A52T	1.000 (probably damaging)	0.789/0.418	Neutral/Damaging	0.0097	0.00058	-
**m.3635G>A**	ND1	S110N	0.999 (probably damaging)	0.873/0.493	Deleterious/Damaging	0	0.00027	-
**m.10663T>C**	ND4L	V65A	0.946 (probably damaging)	0.604/0.694	Deleterious/Damaging	0	0.00003	-
**m.11778G>A**	ND4	R340H	0.999 (probably damaging)	0.919/0.494	Deleterious/Damaging	0.0097	0.0034	-
**m.14484T>C**	ND6	M64V	0.993 (probably damaging)	0.618/0.787	Neutral/Damaging	0.0026	0.00146	-

**Table 4:** Additional, non-synonymous mutations revealed in LHON cases. All these mutations still have no the status of “primary LHON mutations”. A MutPred 1.2 score > 0.75 and a Mutpred 2 score > 0.50 would suggest pathogenicity.

Mutation	Protein-Coding Region of mtDNA	Amino Acid Substitution	PolyPhen–2 Score	MutPred 1.2/2 Score (Cutoff 0,75/0,50)	PROVEAN /SIFT Prediction	Frequency in General Population (mtDB)	Frequency in General Population (HmtDB)	Family
m.6261G>A	CO1	A120T	0.998 (probably damaging)	0.491/0.324	Neutral/Tolerated	0.0048	0.00553	L01
m.8875T>C	ATP6	F117L	0 (benign)	0.251/0.429	Neutral/Tolerated	0.0007	0.001276	L03
m.9921G>A	CO3	A239T	0.009 (benign)	0.543/0.624	Deleterious/Damaging	0.0011	0.00082	L12
m.15468C>T	CYB	T241M	0.890 (possible damaging)	0.245/0.079	Neutral/Tolerated	0.0004	0.00043	L28
m.8551T>C	ATP6	F9L	0.976 (probably damaging)	0.676/0.418	Deleterious/Damaging	0.0007	0	L30
m.14582A>G	ND6	V31A	0.003 (benign)	0.245/0.181	Neutral/Tolerated	0.0086	0.00571	L40
m.8400T>C	ATP8	M12T	0 (benign)	0.504/0.118	Neutral/Tolerated	0.0011	0.00052	L43
m.9444C>T	CO3	R80W	0.999 (probably damaging)	0.875/0.586	Deleterious/Damaging	0	0	
m.4639T>C	ND2	I57T	0.001 (benign)	0.297/0.047	Neutral/Tolerated	0.0082	0.00395	L50
m.8412T>C	ATP8	M16T	0.711 (possible damaging)	0.677/0.542	Deleterious/Tolerated	0	0.00039	
m.15077G>A	CYB	E111K	0.992 (probably damaging)	0.684/0.331	Deleterious/Damaging (low confidence)	0.0007	0.00213	
